# Haemato-oncology and burnout: an Italian survey

**DOI:** 10.1038/sj.bjc.6604270

**Published:** 2008-02-19

**Authors:** C Bressi, S Manenti, M Porcellana, D Cevales, L Farina, I Felicioni, G Meloni, G Milone, I R Miccolis, M Pavanetto, L Pescador, M Poddigue, L Scotti, A Zambon, G Corrao, G Lambertenghi-Deliliers, G Invernizzi

**Affiliations:** 1 Psychiatric Clinic, Milan State University, Fondazione IRCCS Ospedale Maggiore Policlinico, Via F. Sforza 35, Milano 20122, Italy; 2 Dipartimento di Ematologia e Oncologia – Ospedale Centrale, via Boheler 5, Bolzano 39100, Italy; 3 UO Ematologia – Istituto Nazionale dei Tumori, via Venezian 1, Milano 20133, Italy; 4 Università di Perugia – Ospedale Santa Maria della Misericordia, via S. Andrea delle Fratte, Perugia 06156, Italy; 5 Dipartimento di Biotecnologie Cellulari ed Ematologia, Università La Sapienza, via Benevento 6, Roma 00161, Italy; 6 Divisione di Ematologia e di Trapianto di Midollo Osseo, Ospedale Ferrarotto, via Citelli 6, Catania 95124, Italy; 7 Clinica Ematologia-TMO, Università di Milano – Bicocca, Ospedale San Gerardo Nuovo, via Pergolesi 33, Monza 20052, Italy; 8 Dipartimento di Ematologia, Ospedale Umberto I, via Circonvallazione 50, Mestre 30173, Italy; 9 UO Ematologia e Centro Trapianti Midollo Osseo, Ospedale S. Camillo-Forlanini – Circ.ne Gianicolense, Roma 87-00152, Italy; 10 Struttura complessa di Ematologia, Ospedale Oncologico Armando Businco, via Jenner 9, Cagliari 09121, Italy; 11 Dipartimento di Statistica, Università di Milano Bicocca, p.zza dell’Ateneo Nuovo 1, Milano 20126, Italy; 12 Dipartimento di Ematologia e Oncologia, Fondazione IRCCS Ospedale Maggiore Policlinico, via Sforza 35, Milano 20122, Italy

**Keywords:** burnout, job stress, job satisfaction, haematology, oncology

## Abstract

This cross-sectional survey aimed to evaluate the prevalence of burnout and estimated psychiatric disorders among haemato-oncology healthcare professionals in Italy. The aspects of work that respondents perceive as stressful and satisfying have also been examined. The assessments were made using the Maslach Burnout Inventory (MBI), General Health Questionnaire and a study-specific questionnaire. Logistic regression models were applied to show associations between different sources of work-related stress and burnout. Three hundred and eighty-seven out of 440 (87.95%) returned their questionnaires. The scores on MBI subscales indicate a high level of emotional exhaustion in 32.2% of the physicians and 31.9% of the nurses; a high level of Depersonalisation in 29.8 and 23.6%, respectively; and a low level of personal accomplishment in 12.4 and 15.3% respectively. The estimated prevalence of psychiatric disorders was 36.4% in physicians and 28.8% in nurses. Statistical analysis confirmed age, sex, personal dissatisfaction, physical tiredness and working with demanding patients to be associated with burnout. In conclusion, haemato-oncology healthcare professionals report a level of burnout and estimated psychiatric morbidity comparable to other oncological areas. Knowledge of the mechanisms of burnout and preventing and dealing with them is therefore a fundamental requirement for the improvement of quality in health services and job satisfaction.

The term ‘burnout’ was first introduced by Freudenberger (1974) to describe a state of work-related psychological distress in healthcare employees, due to prolonged contact with suffering and dying people. According to C. Maslach (Maslach, 1976; Maslach *et al*, 1996), it is characterised by the three related, but independent, components of emotional exhaustion (EE), depersonalisation (DP) and reduced personal accomplishment (PA), which can be investigated by means of the Maslach Burnout Inventory (MBI). Some somatic symptoms such as tiredness, headaches, sleeplessness and gastrointestinal troubles may often be found. It has been observed among help professionals such as physicians, nurses, social workers, teachers, law enforcement workers and fire fighters (Felton, 1998).

Burnout has been studied in different areas of Medicine such as Oncology, Intensive and Palliative Care Units, and Psychiatric Services, suggesting a higher prevalence among general and hospital practitioners who face chronic or life-threatening diseases (Olkinuora *et al*, 1990; Catalan *et al*, 1996; Ramirez *et al*, 1998; Thomsen *et al*, 1999; Grassi and Magnani, 2000; Graham and Ramirez, 2002; Fothergill *et al*, 2004; Embriaco *et al*, 2007).

It seems to be due to a gradual shift in the balance between individual resources and system requirements, which is attributable to the worker's background and personality characteristics, work organisation and cultural causes. Following individual factors have been found to be associated with burnout: being young, single and female, early life events, family or past history of psychiatric disorder, personality traits of neuroticism and self-criticism, lack of communication and management skills, surface learning style and surface-disorganised approach to work, perceived overwork, low pay and lack of personal or vacation time and extra-work stress (Mount, 1986; Olkinuora *et al*, 1990; Whippen and Canellos, 1991; Ramirez *et al*, 1995, 1996; Deary *et al*, 1996; Wall *et al*, 1997; Weinberg and Creed, 2000; McManus *et al*, 2002; Delva *et al*, 2002). Organisational factors associated with burnout were found to be overload, time urgency, practice routine, increasing managerial burden, inadequate staffing, long working hours and poor resources (Ramirez *et al*, 1996; Arnetz, 1997; Grunfeld *et al*, 2000). Cultural and social context is also a determinant factor for physicians’ perceived work-related stress (Smith, 2001).

Burnout results in a deterioration of the personal and professional life of the caregivers, emerging problems of marital and friendship conflicts, increased prevalence of mood and anxiety disorders, substance abuse and even suicide (BMA, 1993; Caplan, 1994; Blenkin *et al*, 1995; Wall *et al*, 1997; Tyssen and Vaglum, 2002). At an organisational level, burnout syndrome seems to be associated with negative outcomes, such as absenteeism, high staff turnover rates and reduced productivity (McCue, 1982; Wilkinson, 1993; Cordes and Dougherty, 1993), all of which affect the efficiency of the healthcare system, reducing the quality of care delivered to patients causing dissatisfaction among patients, as pointed out by Brook and McGlynn (1996). Treating patients in an impersonal way, with cynicism or even avoiding them is probably the most prominent symptom that differentiates burnout from other types of work-related stress.

Haemato-oncological patients are often affected by incurable chronic cancers, such as leukaemia, lymphoma and multiple myeloma, which reduce their quality of life and require debilitating and life-threatening therapies such as bone marrow transplantation (BMT). Patients are subjected to great psychosocial distress, even in the form of psychiatric disorders (Haberman, 1995; Prieto *et al*, 2002; Fritzsche *et al*, 2003). The daily exposure to suffering and loss can lead the haemato-oncological physicians and nurses to experience strong emotions, such as feelings of inadequacy, grief, anger, disillusionment and frustration, sense of failure, desire to avoid patients and detachment to work (Meier *et al*, 2001). Many studies have investigated prevalence of burnout (30–50%) and psychiatric morbidity (about 25%) among large cohorts of oncological physicians (Whippen and Canellos, 1991; Ramirez *et al*, 1995, 1996; Grunfeld *et al*, 2000), whereas there is little evidence of the extent of these problems on BMT units.

Kiss (1994) described the experience in 4 years of continuous psychosomatic liaison activity at the BMT unit at the University Hospital in Basel, indicating that constant and reliable team support helps to stabilise the team, adding to its inner security. In the UK, Molassiotis *et al* (1995) investigated psychological stress among 129 nurses and 26 doctors from 16 BMT centres. Half were emotionally exhausted, 80% reported feelings of low PA and a significant proportion, particularly medical staff, had marked feelings of DP. Kelly *et al* (2000) argued that the role of nursing needs to be reconceptualised within BMT to allow key humane concerns such as suffering and the emotional labour of care to be explored and better understood.

The aims of this survey were the following: (a) to evaluate, in the Italian scenario, the prevalence of burnout and estimated psychiatric morbidity among a population of hospital physicians and nurses, who worked in haemato-oncological centres equipped with a BMT facility; (b) to show whether there were differences in prevalence among the two different occupations on this topic; (c) to reveal any correlations with sociodemographic factors and sources of job stress and satisfaction.

## MATERIALS AND METHODS

This multicentre survey was coordinated by the Psychiatric Clinic, Fondazione IRCCS, Ospedale Maggiore Policlinico, Milan (Director: Professor G Invernizzi) in collaboration with the Marcora Bone Marrow Transplantation Centre of the same hospital (Director: Professor Lambertenghi-Deliliers). Eighteen national public haematology centres equipped with internal BMT facilities were invited to participate. All centres collaborate with the reference centre inside the European Group for Blood and Marrow Transplantation.

Eight centres refused to participate: five were not interested to the topic and three cited concern about insufficient time and organisational difficulties. The nine centres that agreed (making a total of ten including Marcora BMT Centre) provided different numbers of participants, and they are representative of the main geographical areas in Italy (Bolzano, Catania, Cagliari, Milano, Monza, Perugia, Roma, Venezia).

The study began in April 2005 and ended in October 2005. All of the centres were sent a set of test materials for each one of their physicians and nurses and were given a time limit of 2 weeks to return it with a guarantee of anonymity. The forms were then sent to, examined and processed by the coordinating centre (Scientific leader: Professor C Bressi) and those not correctly completed were excluded.

### Assessment instruments

Each subject completed the following questionnaires:

1. Maslach Burnout Inventory: The MBI is a 22-item self-completed questionnaire with the following subscales: ‘EE’ defined as a lack of the energy necessary to confront the working day, accompanied by a sensation of being emptied and having exhausted all of one's emotional resources, with common feelings of apathy and detachment in relation to work. ‘DP’ defined as professional behaviour characterised by detachment, coldness, boredom, irritation and hardness to the point of hostility, leading to the subject effectively withdrawing from his/her work and relationships with the people they are aiding, whose needs they tend to underestimate. Reduced ‘PA’ refers to feelings of competence, efficiency, productivity and successfully completing one's work. The subject is asked to reply to each question on the basis of a Likert scale measuring the frequency of the event over time (from 0=never to 6=every day). These subscales are considered ‘high’, ‘average’ or ‘low’ according to predetermined cut-off scores based on normative data. Scores are considered ‘high’ if they are in the upper third of the normative distribution, ‘average’ if they are in the middle one, ‘low’ if they are in the lower third. The Italian validation of the questionnaire established the following ranges: high EE ⩾24, DP ⩾9, PA ⩾37; average EE=15–23, DP=4–8, PA=30–36; low EE ⩽14, DP ⩽3, PA ⩽29. The Italian normative sample of 748 Italians working in the healthcare professions gave EE=20.18±11.29; DP=7.03±5.9; PA=32.52±8.66 (Sirigatti and Stefanile, 1993). Cronbach's α coefficient was used to test the internal consistency reliability for each MBI subscale. The raw (standardised) values were 0.778 (0.924) for EE, 0.748 (0.784) for DP and 0.729 (0.786) for PA.

2. The 12-item version of the General Health Questionnaire (GHQ-12) is a screening instrument that is mainly used in community medicine and occupational settings (Goldberg and Williams, 1988; Goldberg *et al*, 1997). It consists of 12 questions designed to investigate 12 frequent psychiatric symptoms (e.g. depression, sleep disturbances). Each item is scored 0 for the answer ‘less or not more than usual’ or 1 for the answer ‘more or much more than usual’ (range 0–12); the sum of each item score gives an individual score. Individuals scoring 4 or more are estimated to have psychiatric morbidity (sensitivity: 75%, specificity: 74%, positive predictive value: 50%; negative predictive value: 90%), according to the Italian validating study (Piccinelli *et al*, 1993) and this threshold was applied.

3. Study-specific questionnaire: This is a questionnaire designed specifically for the study. Items were derived from the international literature and a pilot study involving the physicians and nurses of the coordinating centre. In addition to sociodemographic variables, such as age, gender, occupation and marital status, it included 17 items about sources of stress and 11 items about sources of satisfaction scored 0–1 for ‘yes’ or ‘no’.

A global rating of satisfaction was obtained by asking health care professionals: ‘how satisfying do you find your work?’ on scales of 0–4 (‘not at all satisfied’ to ‘very satisfied’). Finally, perceived quality of clinical, managerial and communicational training was assessed as ‘adequate’ or ‘not adequate’.

### Statistical analysis

Differences between physicians and nurses were evaluated in terms of sociodemographic characteristics, components of stress related to patients, components of stress related to job, type of training received, prevalence of burnout (as measured by the MBI) and prevalence of psychiatric morbidity (as measured by the GHQ-12) by means of *χ*^2^-test.

The presence of deviation of mean score of the MBI subscales from the ones of Italian normative sample were assessed using *z*-tests.

Correlations between the MBI subscales scores and GHQ scores were estimated using Spearman's correlation coefficients.

Differences in the prevalence of estimated psychiatric disorder and burnout were assessed using *χ*^2^-test.

Multiple logistic regression models were fitted on a unique cohort of healthcare staff to estimate the association between different sources of stress and sociodemographic characteristics with burnout. The MBI subscales were dichotomised considering presence of burnout values ⩾24 for EE, ⩾9 for DP and ⩽29 for PA. The selection of covariates was divided in two steps. In the first step, univariate logistic models were fitted for each source of stress and sociodemographic characteristic. The aim of this first step was to identify the variables significantly related with the presence of burnout according to the Wald χ^2^ test. In the second step, each variable related to burnout in at least one MBI subscale were included in the multivariate model according to a forward stepwise approach. The likelihood ratio test was used to evaluate the improvement in the goodness of fit to select the variables to include in the final model. To evaluate whether the type of healthcare professional modifies the effect of variables, we included for each selected covariate the interaction term with profession. The measures of association were expressed as Odds ratio (OR) and corresponding 95% confidence intervals (95% CI) Data were analysed using SAS Version 8.2 (SAS Institute, Cary, NC). All tests with a *P*-value <0.05 were considered statistically significant.

## RESULTS

Four hundred and forty envelopes were delivered to the ten participating centres, and 387 were returned; the response rate from individual centres varied from 76 to 100% (mean 87.95%). Out of the returned questionnaires 37 were only partially completed, leaving 350 (79.54%) in the analysis. Respondents were 121 haematologists and 229 professional nurses. Table 1 shows sociodemographic characteristics of the nursing (*n*=229) and medical sample (*n*=121).

### Burnout and estimated psychiatric morbidity

Table 2 shows the prevalence of burnout components (EE, DP, PA) and GHQ scores. No significant difference was found in the estimated prevalence of burnout and GHQ between nurses and physicians. General Health Questionnaire correlated directly with EE (*r*=0.562 *P*-value < 0.001) and DP (*r*=0.176 *P*-value 0.01), inversely with PA (*r*=−0.333 *P*-value <0.001).

The mean scores of EE (19.94±12.74 among phsysicians, 18.30±11.92 among nurses), PA (37.52±8.6 and 37.15±7.05) and DP (6.07±5.54 and 5.54±5.33) were not significantly different from those of the Italian normative sample.

### Job stress and satisfaction

According to the results of study-specific questionnaire, significantly more physicians (64.5%) than nurses (46.3%) defined themselves as being ‘stressed by work’ (*χ*^2^=11.39 *P*-value 0.001), although significantly more nurses (73.9 *vs* 26.1%, *χ*^2^=6.956 *P*-value 0.008) said that their work could be a risk for their health.

Table 3 shows the distribution of nurses and physicians according to each source of stress related to job, source of stress related to patients and judgement of training received.

Compared with nurses, physicians seemed to be more stressed by the inadequacy of healthcare facilities (*P*-value=0.005), lack of free time (*P*-value <0.001), ethical and moral problems (*P*-value=0.001), and seemed to have a better judgement on the communication training received (*P*-value=0.002). Nurses seemed to be more stressed by physical tiredness (*P*-value=0.025) and working with young patients (*P*-value=0.004) and seemed to have a better judgement than physicians of the managerial training (*P*-value=0.002).

The results relating to the area of job satisfaction showed that about 30% of the physicians and nurses were very satisfied, 60% quite satisfied and only 10% not very or not at all satisfied. Majority of the physicians and nurses (82% and 86%, respectively) would repeat their occupational choice, and 74 and 70%, respectively, would repeat the choice to work with oncological patients. The reasons for professional satisfaction were not significantly different between the physicians and nurses, the most frequently indicated being interpersonal contact with the patients (about 80%), personal satisfaction (55%), personal ideals (55.4% of the physicians *vs* 36.7% of the nurses) and good relationships with colleagues (40%). Less than 30% were religious faith, success, income and research.

Healthcare professionals’ judgement about the adequacy of the training received in different work areas is reported in Table 3.

### Logistic regression

**Figure 1:**
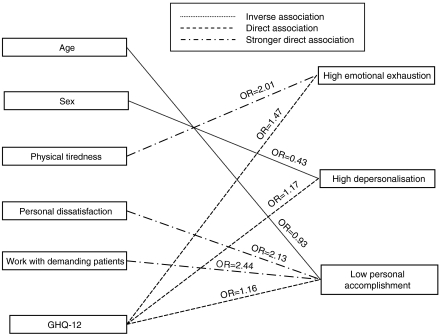
Relationship between prevalence of burnout and sociodemographic characteristics, estimated psychiatric morbidity and sources of stress.

Odds ratio and the corresponding 95% confidence intervals among MBI subscale and sociodemographic characteristics, sources of stress and GHQ were showed in Table 4. High emotional exhaustion seemed to be directly associated with physical tiredness (OR 2.01, 95% CI 1.12–3.61) and GHQ (OR 1.47, 95% CI 1.32–1.63), high DP seemed to be associated with sex (OR 0.43 95%, CI 0.24–0.80) and GHQ (OR 1.17, 95% CI 1.07–1.28). Low personal accomplishment seemed to be inversely related to age (OR 0.93 95%, CI 0.89–0.98), losing patient (OR 0.46 95%, CI 0.22–0.97) and lack of free time (OR 0.43 95%, CI 0.20–0.93), whereas a direct association seemed to be found for personal dissatisfaction (OR 2.13 95%, CI 1.04–4.36), work with demanding patients (OR 1.16 95%, CI 1.07–5.60) and GHQ (OR 1.16 95%, CI 1.04–1.30) (Figure 1). No interaction term tested was significant.

## DISCUSSION

The level of burnout, particularly in the EE scale, was in the range of 30–50% prevalence recorded in other similar studies in the oncological area (Ramirez *et al*, 1995, 1996; Grunfeld *et al*, 2000) and MBI subscales mean scores were not significantly different from those of the Italian normative sample. Comparison with a representative Italian sample (Grassi and Magnani, 2000) of 328 hospital physicians and general practitioners revealed small differences: high EE was 27%, high DP was 25% and low PA was 13%. In a recent study of 242 Italian hospital nurses (Tabolli *et al*, 2006), the mean values of the three MBI subscales were similar to those found in this survey (EE=18.4, DP=3.9 and PA=38.1). A study conducted in 16 BMT centres in the UK (Molassiotis *et al*, 1995) showed that the haematologists had worse burnout subscale scores than the nurses: high EE 33.4 *vs* 12.9% (*P*<0.05), high DP 12.5 *vs* 2.4% (*P*<0.05) and low PA 33.4 *vs* 31.5%. In comparison with these data, the Italian physicians showed equal EE and higher DP, and the nurses showed very high EE and DP. Low PA instead was less representative.

Prevalence of estimated psychiatric morbidity was not significantly higher in physicians (36.4%) than in nurses (28.8%), but both were higher than the general Italian population (22.3%; Politi *et al*, 1994) or the general and hospital physicians (20.3 and 24.3%; Grassi and Magnani, 2000). The previously mentioned study on hospital nurses (Tabolli *et al*, 2006) found 34% of estimated psychiatric cases. In England, the situation appeared to be similar, with a 22–30% prevalence of psychiatric disorder among physicians, significantly higher than the 18% recorded in the general population (Blenkin *et al*, 1995; Ramirez *et al*, 1995, 1996). Molassiotis *et al* (1995), in the aforementioned study, using the HADS scale found an anxiety disorder in 11% of the cases, and symptoms of clinical depression in 3.8% of the physicians and 0.8% of the nurses.

In terms of the MBI and GHQ results, there were no significant differences between physicians and nurses. This was an interesting finding, because the two samples were different in background and job characteristics, which definitely reflected a historical and cultural inheritance. The nurses were mainly young women, whereas the physicians were older and more frequently male. Daily contact time with patients was much higher for nurses. Physicians were responsible for the therapeutic process and the major clinical and ethical decisions, often dealing with communicating bad news, whereas nurses spent more time with the patients attending to their practical demands and looking after their bodily and spiritual needs. Moreover, although physicians have definitely chosen their profession, in Italy nurses have the possibility to change their position within the hospital.

Despite these differences, the two groups responded similarly to work-related stress. The physicians complained of job stress more frequently than the nurses, but the nurses appeared to be more aware of the work-related risk to their health. With some significant differences in the two groups, the main perceived sources of stress would seem to be the working environment and its organisation, which lead to an excessive workload in terms of both time and physical energy. Although both samples indicated an excessive workload as the primary cause of stress, the physicians complained particularly about inadequate organisation and lack of free time, and the nurses about physical tiredness and low salary; work climate was recognised to be a possible source of job stress and satisfaction. Losing the patients did not appear to be a significant source of stress. These data seemed to confirm the findings of another study (Ramirez *et al*, 1996) in which the causes of stress were classified as follows in order of importance: an excessive workload, bad organisation and poor resources, having managerial responsibilities and, finally, having to face the suffering of the patients. Many other authors have highlighted the role of perceived workload and organisational problems in the development of burnout (Cordes and Dougherty, 1993; Barni *et al*, 1996; Arnetz, 1997; Grunfeld *et al*, 2005). Over recent years, Maslach has forcefully argued that the main causes of the development of burnout are related more to the working environment than the individual, and that these aspects should be worked on in a preventive perspective (Maslach and Leiter, 1998). McManus *et al* (2004) also found in a longitudinal study that doctors who reported more stress, burnout and job dissatisfaction complained of excessive workload and an unsupportive work climate.

Although many of the physicians and nurses showed the presence of burnout and acknowledged being stressed at work, PA was low only in 12–15% of cases, and a very high percentage of them declared that they were very or quite satisfied with their work (90%) and would like to repeat the same choice of occupation (about 80%). The major source of motivation was their relationship with patients (80%). This situation was similar to other contexts (Ramirez *et al*, 1996; Arnetz, 1997) and shows how attachment to work and patients hardly declines, even in burnout. Ramirez hypothesised that stress may be ‘double-edged’ in the sense that a task done badly was a source of stress, but the same job done well was a source of satisfaction.

At the same time, it has been reported that highly motivated health professionals with intense investment in their profession are at greater risk for the development of burnout (Leiter and Maslach, 2005).

Physicians judged their training to be adequate in relation to clinical and communicational tasks, but not in relation to managerial duties, thus clearly highlighting what we believed to be a gap between university training and working reality. Their judgements only partially overlapped with those obtained by Ramirez *et al* (1995), who found that the percentages of adequacy for the three areas were 90, 45 and 22% (clinical, communicational, managerial tasks, respectively). Investing energy in good training may therefore reduce job stress (Fallowfield *et al*, 2002; Travado *et al*, 2005).

Despite the cross-sectional nature of the survey that did not allow to define causal relationship, the logistic regression suggested that some organisational and personal factors were associated with burnout subscales, without significant differences between physicians and nurses. The age increase was found to be weakly associated with higher PA. Women appeared to be less exposed to DP. Physical tiredness was associated with high EE, while working with demanding patients, in which continuative care was needed, was strongly associated with low PA.

High GHQ level was associated with all MBI subscales, especially with EE. The literature is very clear on the subject: the development of burnout is associated with psychiatric morbidity (Cordes and Dougherty, 1993; Ramirez *et al*, 1996; Weinberg and Creed, 2000). McManus *et al* (2002), in a longitudinal study, found a circular relationship in which EE made doctors more psychologically distressed (as measured by GHQ), and distress made doctors more emotionally exhausted. The other components of burnout also affected GHQ levels, although neither was itself caused by high GHQ. Molassiotis *et al* (1995) found EE associated with an excessive workload, anxiety, DP and little job satisfaction, and, according to Maslach *et al* (1996), EE is the most responsive scale to the organisational environment and social interactions that characterise human service work.

However, there are some limitations to this study:

Firstly, the cross-sectional design of the survey does not allow us to establish a causal relationship between the variables investigated by means of study-specific questionnaire and the prevalence of burnout and estimated psychiatric morbidity. Prospective cohort design may be used to really understand which factors predict burnout.

Secondly, no standardised interviews were conducted to establish actual diagnoses of psychiatric disorders. It is well known that psychometric tests cannot replace a formal psychiatric evaluation and subjects with a high GHQ-12 score should be evaluated by a psychiatrist.

Furthermore, using only the self-reported questionnaire may have impacted results (Graham and Ramirez, 2002). As suggested by Reid *et al* (1999), to better understand how sources of stress influence staff well-being and views of their job, a qualitative study may be conducted to allow consideration of how burnout and poor mental health might be prevented or improved among haemato-oncological staff. Another limitation is related to the fact that 8 out of 18 haematology centres refused to participate in the survey. Non-responders have been contacted to ask the main reasons that they did not participate in the study. Three centres cited concern about insufficient time and organisational problems, which may suggest that they presented higher levels of burnout and dissatisfaction than respondents. On the other hand, five centres were not interested in the topic, so there is the possibility, according to Renzi *et al* (2005), that totally satisfied haemato-oncological healthcare professionals did not complete the survey, because they felt they had nothing to complain about.

In conclusion, this cross-sectional descriptive study investigated the prevalence of burnout and psychological distress among haemato-oncological healthcare professionals representative of the main geographical areas in Italy. Results indicated a prevalence of burnout similar to prevalence recorded in other international similar studies conducted in the oncological area. According to GHQ-12 scores, 36.4% of the physicians and 28.8% of the nurses could be assessed as having a psychiatric diagnosis or disease upon interview.

Logistic regression suggested that sociodemographic characteristics and environmental factors were associated with MBI subscales.

Further investigations on larger samples and with prospective design are warranted.

## Figures and Tables

**Table 1:** Distribution of 121 physicians and 229 nurses according to sociodemographic characteristics

	**Physicians *N* (%)**	**Nurses *N* (%)**	**χ^2^ (*P*-value)**
*Sociodemographic characteristics*			
Age (years)			
Mean±s.d.	39.17±10.67	37.14±7.51	1.86 (0.064)^a^
Gender			
Male	50 (41.32)	62 (27.07)	7.39 (0.007)
Female	71 (58.68)	167 (72.93)	
Marital status			
Single	53 (43.80)	71 (31.00)	7.48 (0.024)
Married/cohabiting	63 (52.07)	136 (59.39)	
Divorced/separated	5 (4.13)	22 (9.61)	
Years working with oncological patients			
Mean±s.d.	12.27±9.66	8.70±6.80	3.62 (0.0004)^a^
Patient contact time			
Less than 50%	45 (37.19)	42 (18.34)	15.06 (<0.001)
More than 50%	76 (62.81)	187 (81.66)	

a *t*-test.

**Table 2:** Prevalence of burnout and estimated psychiatric morbidity in physicians and nurses

	**Physicians *N* (%)**	**Nurses *N* (%)**	***χ*^2^ (*P*-value)**
*Emotional exhaustion* a			
High (⩾24)	39 (32.2)	73 (31.9)	5.52 (0.06)
Moderate (15–23)	39 (32.2)	50 (21.8)	
Low (⩽14)	43 (35.6)	106 (46.3)	
			
*Depersonalisation* a			
High (⩾9)	36 (29.8)	53 (23.1)	1.93 (0.38)
Moderate (4–8)	31 (25.6)	68 (29.7)	
Low (⩽3)	54 (44.6)	108 (47.2)	
			
*Personal accomplishment* a			
High (⩾37)	75 (62.0)	143 (62.5)	0.84 (0.66)
Moderate (30–36)	31 (25.6)	51 (22.3)	
Low (⩽29)	15 (12.4)	35 (15.3)	
			
*GHQ-12*			
⩾4	44 (36.4)	66 (28.8)	2.09 (0.15)
<4	77 (63.6)	163 (71.2)	

GHQ=General Health Questionnaire

a Cut-off identified according to the Italian validation of the questionnaire.

**Table 3:** Distribution of 121 physicians and 229 nurses according to sources of stress related to job and patients and judgement of training received

	**Physicians *N* (%)**	**Nurses *N* (%)**	***χ*^2^ (*P*-value)**
*Sources of stress related to job*			
Excessive workload	72 (59.50)	143 (62.45)	0.29 (0.591)
Inadequacy of healthcare facility	70 (57.85)	96 (41.92)	8.06 (0.005)
Lack of free time	70 (57.85)	75 (32.75)	20.56 (<0.001)
Physical tiredness	54 (44.36)	131 (57.21)	5.03 (0.025)
Negative relationship with superiors	51 (42.15)	83 (36.24)	1.17 (0.280)
Low salary	42 (34.71)	110 (48.03)	5.72 (0.017)
Negative relationship with colleagues	43 (35.54)	100 (43.67)	2.17 (0.141)
Losing patients	40 (33.06)	89 (38.86)	1.15 (0.284)
Personal dissatisfaction	43 (35.54)	76 (33.19)	0.19 (0.659)
Excess of responsibility	50 (41.32)	74 (32.31)	2.81 (0.094)
Ethical and moral problems	42 (34.71)	44 (19.21)	10.26 (0.001)
			
*Sources of stress related to patients*			
Work with young patients	57 (47.11)	145 (63.32)	8.53 (0.004)
Work with terminally ill patients	56 (46.28)	106 (46.29)	0.00 (0.999)
Work with suffering patients	52 (42.98)	89 (38.86)	0.56 (0.456)
Work with patients’ families	42 (34.71)	76 (33.19)	0.082 (0.774)
Work with demanding patients	20 (16.53)	33 (14.41)	0.28 (0.599)
Work with patients with life-threatening diseases	41 (33.88)	62 (27.07)	1.77 (0.184)
			
*Judgement of training received*			
Adequate clinical training	108 (90)	201 (93.49)	1.31 (0.253)
Adequate communication training	103 (85.83)	139 (70.20)	10.04 (0.002)
Adequate managerial training	43 (36.44)	110 (54.73)	9.96 (0.002)

**Table 4:** Odds ratios and corresponding 95% confidence intervals according to sociodemographic characteristics, GHQ-12 and sources of stress related to job and patients

	**High EE**	**High DP**	**Low PA**
**Dependent variable**	**OR^a^ (95%CI)**	**OR^a^ (95%CI)**	**OR^a^ (95%CI)**
*Sociodemographic charateristics*			
Age	1.03 (0.99–1.06)	0.98 (0.94–1.01)	0.93^*^ (0.89–0.98)
Sex	0.93 (0.47–1.84)	0.43^*^ (0.24–0.80)	1.58 (0.69–3.63)
Profession	1.15 (0.59–2.26)	0.89 (0.48–1.65)	0.89 (0.38–2.09)
			
*Sources of stress related to job*			
Physical tiredness	2.01^*^ (1.12–3.61)	1.64 (0.94–2.88)	1.00 (0.48–2.07)
Personal dissatisfaction	1.47 (0.82–2.64)	1.49 (0.86–2.57)	2.13^*^ (1.04–4.36)
Losing patients	1.29 (0.72–2.29)	0.75 (0.43–1.33)	0.46^*^ (0.22–0.97)
Low salary	1.35 (0.76–2.41)	1.19 (0.69–2.05)	1.30 (0.64–2.63)
Lack of free time	1.53 (0.85–2.76)	1.41 (0.81–2.47)	0.43^*^ (0.20–0.93)
			
*Sources of stress related to patients*			
Work with terminally ill patients	1.67 (0.95–2.95)	1.28 (0.75–2.20)	1.74 (0.85–3.55)
Work with demanding patients	1.83 (0.87–3.85)	1.54 (0.76–3.11)	2.44^*^ (1.07–5.60)
			
*Psichiatric morbidity*			
GHQ-12	1.47^*^ (1.32–1.63)	1.17^*^ (1.07–1.28)	1.16^*^ (1.04–1.30)
C-index^b^	0.830	0.719	0.798

CI=confidence interval; DP=depersonalisation; EE=emotional exhaustion; GHQ=General Health Questionnaire; OR=odds ratio; PA=personal accomplishment.

^*^P-value <0.05.

a Estimated from multiple logistic model including terms for place of work, patient contact time and marital status.

b C-index provide the predictive power of a logistic regression model.

## References

[bib1] Arnetz BB (1997) Physicians’ view of their work environment and organisation. Psychother Psychosom 66: 155–162 9176910 10.1159/000289127

[bib2] Barni S, Mondin R, Nazzani R, Archilli C (1996) Oncostress: evaluation of burnout in Lombardy. Tumori 82: 85–92 8623514 10.1177/030089169608200119

[bib3] Blenkin H, Deary I, Sadler A, Agius R (1995) Stress in NHS consultants. BMJ 310: 534 7888917 10.1136/bmj.310.6978.534 PMC2548906

[bib4] British Medical Association (1993) The morbidity and Mortality of the Medical Profession – a Literature Review and Suggestions for Future Research. London: BMA

[bib5] Brook RH, Mcglynn EA (1996) Quality of health care. Part 2: measuring quality of care. N Engl J Med 335: 966–970 8782507 10.1056/NEJM199609263351311

[bib6] Caplan RP (1994) Stress, anxiety, and depression in hospital consultants, general practitioners, and senior health service managers. BMJr Med J 310: 524 10.1136/bmj.309.6964.1261 PMC2541798 7888846

[bib7] Catalan J, Burgess A, Pergami A, Hulme N, Gazzard B, Phillips R (1996) The psychological impact on staff of caring for people with serious diseases: the case of HIV infection and oncology. J Psychosom Res 40: 425–435 8736423 10.1016/0022-3999(95)00527-7

[bib8] Cordes CL, Dougherty TM (1993) A review and an integration of research on job burnout. Acad Manag Rev 18: 621–656

[bib9] Deary IJ, Blenkin H, Agius RM, Endler NS, Zealley H, Wood R (1996) Models of job-related stress and personal achievement among consultant doctors. Br J Psychol 87: 3–29 8852018 10.1111/j.2044-8295.1996.tb02574.x

[bib10] Delva MD, Kirby JR, Knapper CK, Birtwhistle RV (2002) Postal survey of approach to learning among ontario physicians: implications for continuing medical education. BMJ 325: 1218 12446540 10.1136/bmj.325.7374.1218 PMC135496

[bib11] Embriaco N, Azoulay E, Barrau K, Kentish N, Pochard F, Loundou A, Papazian L (2007) High level of burnout in intensivists: prevalence and associated factors. Am J Respir Crit Care Med 175: 686–692 17234905 10.1164/rccm.200608-1184OC

[bib12] Fallowfield L, Jenkins V, Farewell V, Saul J, Duffy A, Eves R (2002) Efficacy of a cancer research UK communication skills training model for oncologists: a randomized controlled trial. Lancet 359: 650–656 11879860 10.1016/S0140-6736(02)07810-8

[bib13] Felton JS (1998) Burnout as a clinical enity – its importance in health care workers. Occup Med 48: 237–250 10.1093/occmed/48.4.237 9800422

[bib14] Fothergill A, Edwards D, Burnard P (2004) Stress, burnout, coping and stress management in psychiatrists: findings from a systematic review. Int J Soc Psychiatry 50: 54–65 15143847 10.1177/0020764004040953

[bib15] Freudenberger HJ (1974) Staff burnout. J Soc Issues 30: 159–165

[bib16] Fritzsche K, Struss Y, Stein B, Spahn C (2003) Psychosomatic liaison service in hematological oncology: need for psychotherapeutic interventions and their realization. Hematol Oncol 21: 83–89 12802813 10.1002/hon.711

[bib17] Goldberg DP, Gater R, Sartorius N, Ustun TB, Piccinelli M, Gureje O, Rutter C (1997) The validity of two versions of the GHQ in the WHO study of mental illness in general health care. Psychological Med 27: 191–197 10.1017/s0033291796004242 9122299

[bib18] Goldberg DP, Williams P (1988) A Users Guide to the General Health Questionnaire. Windsor, Berkshire: NFER – Nelson Publishing

[bib19] Graham J, Ramirez A (2002) Improving the working lives of cancer clinicians. Eur J Cancer Care 11: 188–192 10.1046/j.1365-2354.2002.00338.x 12296836

[bib20] Grassi L, Magnani K (2000) Psychiatric morbidity and burnout in the medical professions: an Italian study of general practiotioners and hospital physicians. Psychother Psychosom 69: 329–334 11070446 10.1159/000012416

[bib21] Grunfeld E, Whelan TJ, Zitzelsberger L, William A, Montesanto B, Evans WK (2000) Cancer care workers in Ontario: prevalence of burnout, job stress and job satisfaction. Can Med Assoc J 163: 166–169 10934978 PMC80206

[bib22] Grunfeld E, Zitzelsberger L, Coristine M, Whelan TJ, Aspelund F, Evans WK (2005) Job stress and job satisfaction in cancer care workers. Psychooncology 14: 61–69 15386787 10.1002/pon.820

[bib23] Haberman R (1995) The meaning of cancer therapy: bone marrow transplant as an exemplar of therapy. Semin Oncol Nurs 11: 23–31 7740219 10.1016/s0749-2081(95)80039-5

[bib24] Kelly D, Ross S, Gray B, Smith P (2000) Death, dying and emotional labour: problematic dimensions of the bone marrow transplant nursing role? J Adv Nurs 32: 952–960 11095235

[bib25] Kiss A (1994) Support of the transplant team. Support Care Cancer 2: 56–60 8156258 10.1007/BF00355240

[bib26] Leiter M, Maslach C (2005) A mediation model of job burnout. In Research Companion to Organizational Health Psychology, Antoniou A, Cooper CL (eds), pp 544–564. Cheltenham: Edward Elgar Publishing

[bib27] Maslach C (1976) Burned-out. Hum Behav 5: 16–22

[bib28] Maslach C, Jackson S, Leiter M (1996) Maslach Burnout Inventory Manual, 3rd edn. Palo Alto, CA: Consulting Psychologists Press

[bib29] Maslach C, Leiter M (1998) The Truth About Burnout: how Organizations Cause Personal Stress and what to do About it. San Francisco, CA: Jossey-Bass

[bib30] McCue JD (1982) The effects of stress on physicians and their medical practise. N Engl J Med 306: 458–463 7057844 10.1056/NEJM198202253060805

[bib31] McManus IC, Winder BC, Gordon D (2002) The causal links between stress and burnout in a longitudinal study of UK doctors. Lancet 359: 2089–2090 12086767 10.1016/s0140-6736(02)08915-8

[bib32] McManus IC, Keeling A, Paice E (2004) Stress, burnout and doctors’ attitudes to work are determined by personality and learning style: a twelve year longitudinal study of UK medical graduates. BMC Med 8: 29 10.1186/1741-7015-2-29 PMC516448 15317650

[bib33] Meier D, Back A, Morrison R (2001) The inner life of physicians and care of the seriously ill. JAMA 286: 3007–3014 11743845 10.1001/jama.286.23.3007

[bib34] Molassiotis A, van den Akker OBA, Boughton BJ (1995) Psychological stress in nursing and medical staff on bone marrow transplant units. Bone Marrow Transplant 15: 449–454 7599571

[bib35] Mount BM (1986) Dealing with our losses. J Clin Oncol 4: 1127–1134 3723168 10.1200/JCO.1986.4.7.1127

[bib36] Olkinuora M, Asp S, Juntunen J (1990) Stress symptoms, burnout and suicidal thought in Finnish physicians. Social Psychiatry Psychiatr Epidemiol 25: 81–86 10.1007/BF00794986 2336581

[bib37] Piccinelli M, Bisoffi G, Bon MG, Cunico L, Tansella M (1993) Validity and test-retest reliability of the Italian version of the 12-item general heath questionnaire in general practise: a comparison between three scoring methods. Compr Psychiatry 34: 198–205 8339539 10.1016/0010-440x(93)90048-9

[bib38] Politi PL, Piccinelli M, Wilkinson G (1994) Reliability, validity and factor structure of the 12-item General Health Questionnaire among young males Italy. Acta Psychiatr Scand 90: 432–437 7892776 10.1111/j.1600-0447.1994.tb01620.x

[bib39] Prieto JM, Blanch J, Atala J, Carreras E, Rovira M, Cirera E, Gastó C (2002) Psychiatric morbidity and impact on hospital length of stay among hematologic cancer patients receiving stem-cell transplantation. J Clin Oncol 20: 1907–1917 11919251 10.1200/JCO.2002.07.101

[bib40] Ramirez AJ, Graham J, Richards MA, Cull A, Gregory WM, Leaning MS, Snashall DC, Timothy AR (1995) Burnout and psychiatric disorder among cancer clinicians. Br J Cancer 71: 1263–1269 7540037 10.1038/bjc.1995.244 PMC2033827

[bib41] Ramirez AJ, Graham J, Richards M (1996) Mental health of hospital consultants: the effects of stress and satisfaction at work. Lancet 347: 724–728 8602002 10.1016/s0140-6736(96)90077-x

[bib42] Ramirez AJ, Addington-Hall J, Richards M (1998) ABC of palliative care. The carers. BMJ 316: 208–211 9468691 10.1136/bmj.316.7126.208 PMC2665429

[bib43] Reid Y, Johnson S, Morant N, Kuipers E, Szukler G, Thornicroft G, Bebbington P, Prosser D (1999) Explanations for stress and satisfaction in mental health professionals: a qualitative study. Soc Psychiatry Psychiatr Epidemiol 34: 301–308 10422483 10.1007/s001270050148

[bib44] Renzi C, Tabolli S, Ianni A, Di Pietro C, Puddu P (2005) Burnout and job satisfaction comparing healthcare staff of a dermatological hospital and a general hospital. J Eur Acad Dermatol Venereol 19: 153–157 15752281 10.1111/j.1468-3083.2005.01029.x

[bib45] Sirigatti S, Stefanile C (1993) Adattamento italiano MBI – Maslach Burnout Inventory. Firenze: Organizzazioni Speciali

[bib46] Smith R (2001) Why are doctors so unhappy? BMJ 322: 1073–1074 11337419 10.1136/bmj.322.7294.1073 PMC1120219

[bib47] Tabolli S, Ianni A, Renzi C, Di Pietro C, Puddu P (2006) Soddisfazione lavorativa, burnout e stress del personale infermieristico: indagine in due ospedali di Roma. G Ital Med Lav Ergon 28: 49–52 19031557

[bib48] Thomsen S, Soares J, Nolan P, Dallender J, Arnetz B (1999) Feelings of professional fulfilment and exhaustion in mental health personnel: the importance of organisational and individual factors. Psychoter Psychosom 68: 157–164 10.1159/000012325 10224515

[bib49] Travado L, Grassi L, Gil F, Ventura C, Martins C, Southern European Psycho-oncology study group (2005) Physician-patient communication among Southern European cancer physicians: the influence of psychosocial orientation and burnout. Psychooncology 14: 661–670 15651069 10.1002/pon.890

[bib50] Tyssen R, Vaglum P (2002) Mental health problems among young doctors: an updated review of prospective studies. Harv Rev Psychiatry 10: 154–165 12023930 10.1080/10673220216218

[bib51] Wall TD, Bolden RI, Borrill CS, Carter AJ, Golya DA, Hardy GE, Haynes CE, Rick JE, Shapiro DA, West MA (1997) Minor psychiatric disorder in NHS trust staff: occupational and gender differences. Br J Psychiatry 171: 519–523 9519089 10.1192/bjp.171.6.519

[bib52] Weinberg A, Creed A (2000) Stress and psychiatric disorder in healthcare professionals and hospital staff. Lancet 355: 533–537 10683003 10.1016/S0140-6736(99)07366-3

[bib53] Whippen D, Canellos G (1991) Burnout syndrome in the practice of oncology. Results of a random survey of 1000 oncologists. J Clin Oncol 9: 1916–1920 1919641 10.1200/JCO.1991.9.10.1916

[bib54] Wilkinson P (1993) Mental health problems at work. BMJ 306: 1082–1083 8495151 10.1136/bmj.306.6885.1082 PMC1677531

